# Adult bonobos show no prosociality in both prosocial choice task and group service paradigm

**DOI:** 10.7717/peerj.12849

**Published:** 2022-02-01

**Authors:** Jonas Verspeek, Edwin J. C. van Leeuwen, Daan W. Laméris, Nicky Staes, Jeroen M. G. Stevens

**Affiliations:** 1 Centre for Research and Conservation, Royal Zoological Society of Antwerp, Antwerp, Belgium; 2 Behavioural Ecology and Ecophysiology Group, Department of Biology, University of Antwerp, Wilrijk, Belgium; 3 SALTO, Agro- and Biotechnology, Odisee University College, Brussels, Belgium

**Keywords:** *Pan paniscus*, Free choice, Group experiment, Food-provisioning, Paradigm, Pan universal, Age effect, Zoo-housed, Ecological validity, Great apes

## Abstract

Previous studies reported contrasting conclusions concerning bonobo prosociality, which are likely due to differences in the experimental design, the social dynamics among subjects and characteristics of the subjects themselves. Two hypotheses have been proposed to explain the occurrence of prosociality in animals: the cooperative breeding hypothesis and the self-domestication hypothesis. While the former predicts low levels of prosociality in bonobos because they are non-cooperative breeders, the latter predicts high levels of prosociality because self-domestication has been proposed to select for high levels of tolerance in this species. Here, we presented a group of thirteen bonobos with two platform food-provisioning tasks: the prosocial choice task (PCT) and the group service paradigm (GSP). The latter has so far never been applied to bonobos. To allow for free choice of participation and partner, we implemented both tasks in a group setting. Like in previous PCT studies, bonobos did not choose the prosocial option more often when a group member could benefit *vs* not benefit. In the GSP, where food provisioning is costly, only subadult bonobos showed a limited amount of food provisioning, which was much lower than what was previously reported for chimpanzees. In both experiments, adult subjects were highly motivated to obtain rewards for themselves, suggesting that bonobos behaved indifferently to the gains of group members. We suggest that previous positive food-provisioning prosociality results in bonobos are mainly driven by the behaviour of subadult subjects. The lack of prosociality in this study corresponds to the hypothesis that proactive food provisioning co-occurs with cooperative breeding and suggests that proactive prosociality might not be part of the self-domestication syndrome in bonobos.

## Introduction

Non-human animals (henceforth “animals”) engage in a variety of cooperative interactions, like border patrolling, cooperative hunting, grooming, coalition formation and food sharing (Clutton-Brock, 2009). Prosociality has been suggested as the main facilitating evolutionary driver of cooperation (Fehr & Fischbacher, 2003; Jaeggi, Burkart & Van Schaik, 2010; Silk, 2012; Martin et al., 2021), and has been defined as “any behaviour performed by one individual to alleviate another’s need or to improve their welfare, without the actor necessarily incurring extra costs to provide these benefits” (Amici, Visalberghi & Call, 2014). It has been suggested that humans show unique levels of prosociality, including both reactive prosociality, *i.e*. in response to directed signals of need, and proactive prosociality, *i.e*. in the absence of recipient’s signals of need, because of a unique set of socio-cognitive traits that humans typically possess (Jaeggi, Burkart & Van Schaik, 2010). To gain insight into the evolutionary basis of human prosociality, and to investigate to what extent animals show comparable levels of prosociality, an increasing number of studies has investigated prosociality in animals. Most experimental prosociality studies have focused on primates (reviewed in Marshall-Pescini et al., 2016), but more recent research has also demonstrated prosociality in other species, including rodents (Hernandez-Lallement et al., 2015; Márquez et al., 2015; Schweinfurth & Taborsky, 2018; Lalot et al., 2021b), canids (Quervel-Chaumette et al., 2015; Dale et al., 2016; Dale et al., 2019a; Dale et al., 2019b), cetaceans (Nakahara et al., 2017; Lalot et al., 2021a), corvids (Horn et al., 2016, 2020; Massen, Haley & Bugnyar, 2020), parrots (Krasheninnikova et al., 2019; Brucks & von Bayern, 2020; Laumer et al., 2021) and fish (Satoh et al., 2021). However, other studies found no evidence for prosociality (*e.g*. in chimpanzees (Silk et al., 2005; Vonk et al., 2008), capuchin monkeys (Skerry, Sheskin & Santos, 2011; Drayton & Santos, 2014), cotton-top tamarins (Cronin et al., 2009), meerkats (Amici et al., 2017), corvids (Di Lascio et al., 2013; Lambert et al., 2017; Horn et al., 2021)). These contrasting findings have sparked the interest in which factors could explain the variability in the expression of prosociality. The most extensive comparative prosociality study tested 15 primate species, including humans. The results showed that the level of allomaternal care was the best predictor for the level of proactive prosociality (Burkart et al., 2014 yet, for some nuance see van Leeuwen, 2021), providing evidence for the cooperative breeding hypothesis, *i.e*. “cooperative breeding is accompanied by psychological changes leading to greater prosociality” (Burkart, Hrdy & Van Schaik, 2009). For cooperatively breeding to work, caretakers must proactively seek opportunities to provide food to others (Burkart & van Schaik, 2010). However, other experimental studies showed negative prosociality results in cooperatively breeding species (*e.g*. cotton-top tamarins (Cronin et al., 2009; Stevens, 2010), meerkats (Amici et al., 2017), carrion crows and azure-winged magpies (Horn et al., 2021)), while others also found prosociality in independently breeding species (*e.g*. cichlids (Satoh et al., 2021), long-tailed macaques (Massen et al., 2010), capuchin monkeys (Lakshminarayanan & Santos, 2008), chimpanzees (Warneken & Tomasello, 2006; Melis et al., 2011; van Leeuwen et al., 2021), bonobos (Tan & Hare, 2013; Tan, Ariely & Hare, 2017; Krupenye, Tan & Hare, 2018). This suggests that cooperative breeding is not a prerequisite for prosocial behaviour (Cronin, 2017). Also, it remains debated what social or cognitive effects cooperative breeding might have had on humans and to what extent humans are cooperative breeders Bogin, Bragg & Kuzawa (2014), Thornton & Mcauliffe (2015) and Thornton et al. (2016). Another factor that positively correlated with the level of prosociality was a high level of social tolerance, as measured by the extent of equal access to food by group members in the GSP (Burkart et al., 2014). This fits with the self-domestication hypothesis (Hare, Wobber & Wrangham, 2012; Hare, 2017), which states that prosociality arises as a by-product of selection against aggression and selection for increased tolerance (Wrangham, 2019 but see Sánchez-Villagra & van Schaik, 2019). Both the cooperative breeding hypothesis and the self-domestication hypothesis suggest a link between social tolerance and prosociality, but emphasize different influential factors. Since prosociality is a diverse and flexible trait (Dunfield et al., 2011; Tomasello & Vaish, 2012; Decety et al., 2016), both hypotheses may complement each other to explain different facets of prosocial behaviour rather than being mutually exclusive (Decety et al., 2016; Horn et al., 2020).

The variety of task designs, experimental contexts and reward distributions has led to a large variation in reported prosociality within and between-species (Jensen, 2016a; Marshall-Pescini et al., 2016; Tan & Hare, 2017). Two main types of experimental paradigms have been used to study prosociality in animals: the targeted helping paradigms and food provisioning tasks, each with its own advantages and disadvantages (House et al., 2014; Marshall-Pescini et al., 2016; Tennie, Jensen & Call, 2016; Melis, 2018). Targeted helping paradigms examine whether subjects help others in situations that they cannot resolve on their own (Yamamoto & Takimoto, 2012). They require a certain degree of perspective taking, since subjects have to understand the goals and needs of others to react to it, which could explain why sustainable targeted helping is only found in species with higher levels of such cognitive capacities like great apes, dolphins and elephants (de Waal, 2008; Yamamoto & Takimoto, 2012). In food provisioning tasks, subjects can choose whether to provide food to a recipient or not. The most commonly used food provisioning task is the cost-free prosocial choice task (PCT) (Silk et al., 2005; Jensen et al., 2006), in which subjects can choose between a selfish (1/0) and a prosocial option (1/1). Subjects gain identical payoffs for the same effort in either option, so they should prefer the prosocial option (1/1) if they have a prosocial preference. Two main approaches of the PCT exist: platforms and tokens. In the platform PCT, the two choices are presented on sliding platforms, allowing for a physical choice by the subject. In the token PCT, the two choices are represented symbolically with tokens (de Waal, Leimgruber & Greenberg, 2008; Horner et al., 2011; Amici, Visalberghi & Call, 2014; Dale et al., 2016; Krasheninnikova et al., 2019; Emigh et al., 2020).

Importantly, to allow for comprehensive comparisons of prosociality within and between species, several methodological issues should be considered. A first concern is task complexity. Subjects must understand the contingencies of the task. Some studies have incorporated contingency training phases to ensure task comprehension, but this can lead to overtraining of the subjects and an overestimation of prosocial behaviours (Jensen, 2012; Marshall-Pescini et al., 2016; Tennie, Jensen & Call, 2016). As an alternative, ‘knowledge’ self-tests can be incorporated to test the subject’s understanding of the task. Here, the reward distribution is changed so that subjects can access the better reward by choosing the option with the most food rewards (Burkart et al., 2007; Claidière et al., 2015; Tan, Kwetuenda & Hare, 2015; Jensen, 2016b). The PCT has been suggested to be cognitively too demanding (Burkart & Rueth, 2013; Claidière et al., 2015; Tan, Kwetuenda & Hare, 2015; Melis, 2018). Therefore, an alternative version has been proposed: the group service paradigm (GSP) (Cronin, Schroeder & Snowdon, 2010; Burkart & van Schaik, 2013; Burkart et al., 2014; House et al., 2014; van Leeuwen et al., 2021). In this costly version of the PCT, subjects can choose whether to deliver food only to a partner or to provide no food at all, which has also been referred to as a single-choice (go) task (Jensen, 2016b). The GSP is considered as cognitively less demanding compared to the PCT, because subjects only need to consider the payoff of one food item (instead of three in the PCT) (Burkart et al., 2014). The adjusted payoffs in the GSP essentially turn the standard PCT into an instrumental helping task, since only recipients receive a reward at a low cost for the subject, *i.e*. the energetic cost to pull the handle (House et al., 2014; Tan, Kwetuenda & Hare, 2015). A second limitation of most PCTs and helping studies, is that mainly preselected, compatible or tolerant dyads have been tested, often with the deliberate intent to increase the chance to detect prosociality (*e.g*., Melis, Schneider & Tomasello, 2011; Taglialatela et al., 2020; Nolte & Call, 2021). However, several studies showed the importance of free partner choice in experimental studies (Melis, Hare & Tomasello, 2008; Suchak et al., 2014; Cronin et al., 2017; van Leeuwen et al., 2021). Therefore, experiments that maintain the social group dynamics during testing should be implemented to improve the socio-ecological validity and make biologically relevant conclusions (House et al., 2014; Marshall-Pescini et al., 2016; Cronin et al., 2017; Massen et al., 2019). More recently, the GSP has successfully been implemented in a group setting in a variety of species (Burkart & van Schaik, 2013; Burkart et al., 2014; Horn et al., 2016, 2020; Martin et al., 2021; van Leeuwen et al., 2021), providing additional evidence for the importance of free choice of partner (Martin et al., 2021).

The bonobo, one of humans’ closest living relatives, is a very interesting independently breeding species to investigate the two hypotheses regarding the evolutionary origins of prosociality. Most prosociality studies in great apes have focused on chimpanzees because, among other things, they are more numerous in captivity (Tennie, Jensen & Call, 2016; Melis, 2018). Bonobos are stated to be more peaceful, tolerant and cooperative than chimpanzees (Hare, Wobber & Wrangham, 2012; Maclean, 2016; Hare, 2017) and have been suggested to have undergone the process of self-domestication (Hare, Wobber & Wrangham, 2012; Hare, 2017). Because of this, and their close genetic relatedness to humans, they have been explicitly proposed as an ideal species to study the biological predispositions of prosociality in humans (Maclean, 2016; Hare, 2017; Hare & Yamamoto, 2017; Tan & Hare, 2017). Experimental studies of prosocial behaviour in bonobos, however, are rare and have so far yielded mixed results. Moreover, all these studies have worked with preselected instead of freely interacting dyads. Bonobos showed targeted helping in some (Hare & Kwetuenda, 2010; Tan & Hare, 2013; Tan, Ariely & Hare, 2017; Nolte & Call, 2021), but not all studies (Bullinger et al., 2013; Liebal et al., 2014). They voluntarily handed food items to others (Krupenye, Tan & Hare, 2018), but they did not behave prosocially in a token and platform variant of the PCT (Amici, Visalberghi & Call, 2014; Tan, Kwetuenda & Hare, 2015). To date, bonobos have not been tested on the GSP.

Our aim was therefore to study bonobo prosociality using both the PCT and the GSP in the same bonobo group. This study will be the first to implement the GSP in bonobos, and will contribute to the cross-species comparative framework of prosociality research that employs an identical experimental paradigm (Burkart et al., 2014; Horn et al., 2020). Using both the PCT and GSP approach, we aim to rule out that cognitive limitations resulting from PCT complexity are at the base of negative results in previous bonobo studies (Burkart & Rueth, 2013; Tan, Kwetuenda & Hare, 2015; Melis, 2018). We will, for the first time in bonobos, use the stacked platform PCT rather than the horizontal platform PCT that has been previously used but showed negative results (Amici, Visalberghi & Call, 2014; Tan, Kwetuenda & Hare, 2015). It has been suggested that the horizontal platform PCT task can cause false positives through local enhancement, which can be avoided if the rewards are stacked in a stacked platform PCT (Amici, Visalberghi & Call, 2014; Tan, Kwetuenda & Hare, 2015). Finally, we implement both tasks in group context rather than in preselected dyads, to allow for free choice of participation, resulting in more naturalistic partner choices that will allow for more biologically relevant conclusions (Cronin et al., 2017). Based on the cooperative breeding hypothesis, low to intermediate levels of proactive prosociality are expected in bonobos given that they are an independently breeding species, while the self-domestication hypothesis predicts high levels of prosociality in this species if prosociality indeed co-evolved with selection for higher tolerance and lower aggression.

## Materials and Methods

### Subjects and housing

This study included 13 bonobos (6M:7F) housed at Zoo Planckendael (Belgium) (mean age 15.77 ± 3.12 years, Table 1). They were housed in an indoor enclosure consisting of ten interconnected rooms of varying sizes (from 11 to 100 m^2^ total size: 422 m^2^). No animals were separated for testing. The test apparatus was presented in a central room (35 m^2^), which could be directly accessed from six adjacent rooms. During testing, all animals had access to the apparatus, and could freely move through the entire building. Although we attempted to increase participation by calling the subjects to the central room, participation was always voluntarily. Therefore, individuals varied substantially in the number of trials they participated in (see below). The testing apparatus, baiting process, distribution of food rewards and reward consumption were always visible to the bonobos. To maximise subject’s motivation, we used grape halves as highly favoured food, as this was confirmed to be the preferred food in a previous study (Verspeek & Stevens, 2020). JV videotaped and coded all recordings. No animals were sacrificed or sedated for the purpose of this study. This non-invasive research adhered to the legal requirements of the country in which the research was conducted (Belgium) and was approved by the Scientific Advisory Board of the Royal Zoological Society of Antwerp (EC-3/SGZ(10-12-19)) and the University of Antwerp (Belgium) and endorsed by the European Breeding Program for bonobos. All research complied with the ASAB guidelines (ASAB, 2020).

**Table 1:** Subject information of the prosocial choice task and the group service paradigm.

			Prosocial choice task		Group service paradigm		
Individual	Sex	Age^a^	Number of trials in each condition		Amount of pulls in each condition		Deliveries
			Test	Control	Test	Control	
Hortense^°^	Female	41	–	–	0	0	–
Banya^°^^,^^+^	Female	29	–	–	–	–	–
Vifijo^°^	Male	25	–	–	0	0	–
Djanoa	Female	24	90	20	4	0	0
Zamba^°^	Male	21	–	–	0	0	–
Busira	Female	15	103	19	0	0	–
Kianga	Female	14	118	20	5	0	0
Habari	Male	13	99	20	10	0	0
*Nayoki*	Female	7	50	3	32	1	2
*Mokonzi*	Male	6	12	20	14	1	1
*Kikongo*	Male	5	22	20	38	0	5
*Moko* ^°^ ^,^ ^+^	Male	3	–	–	–	–	–
*Sanza* ^°^ ^,^ +	Female	2	–	–	–	–	–

**Notes:**

Names in *italic*: individuals younger than 8 years were considered as subadults.

a Individual’s age when the study took place, based on the studbook data (J.J.B. Pereboom & J.M.G. Stevens, 2020, unpublished data).

° Individuals that did not pass the training criterion to act as a subject in the PCT.

+ Individuals that did not pass the training criterion to act as a subject in the GSP.

### Setup and procedure

#### Experiment 1: Prosocial choice task

**Figure 1:**
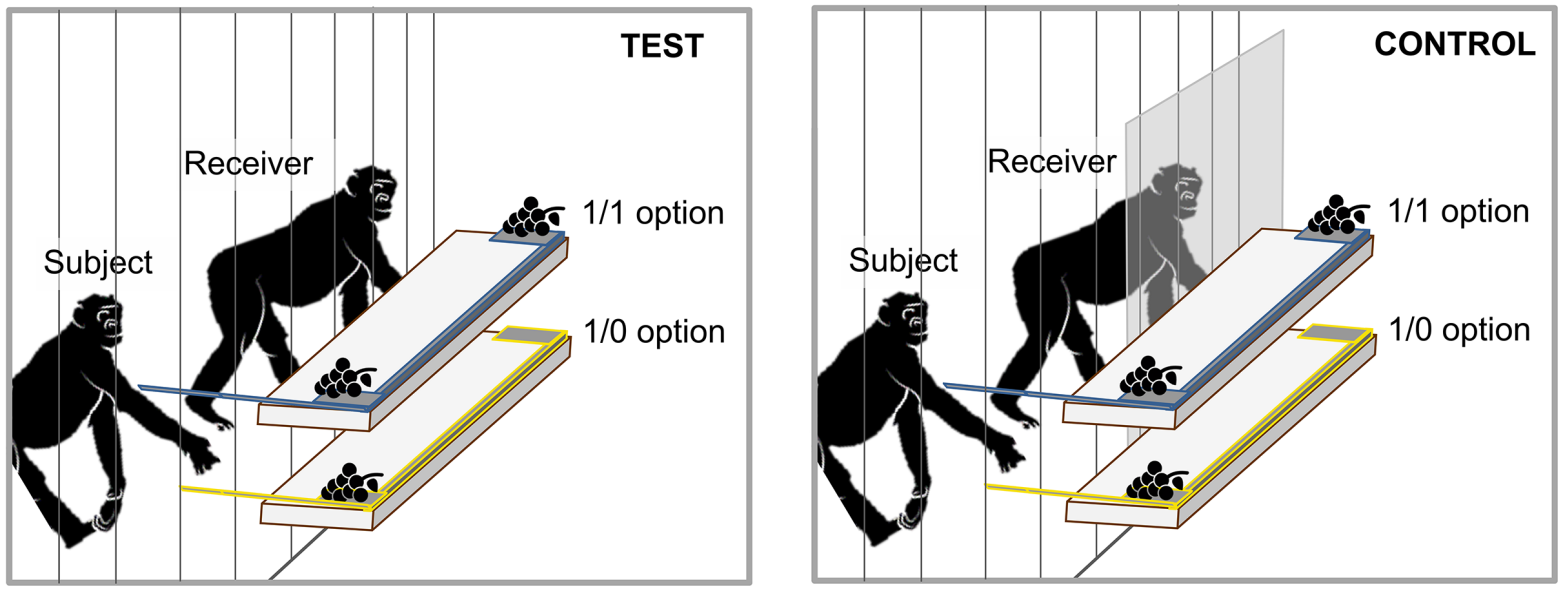
Schematic drawing of the PCT set-up. The handle can be pulled to bring the platform within reach. Each trial, only one bar could be pulled. In the control sessions, the receiver’s reward was unreachable due to a plexiglass panel (online version in colour).

The PCT set-up was based on the original chimpanzee study (Silk et al., 2005): subjects could deliver benefits to group members at no cost for themselves (Silk et al., 2005; Jensen et al., 2006). They could choose between a prosocial option, providing a reward to subject and receiver (1/1) and a selfish option, where only the subject obtains a food reward (1/0). The apparatus consisted of two platforms, each with one handle attached to it, that were stacked on top of each other and which were colour coded to improve discrimination by the subjects (Fig. 1). When a subject pulled a handle, the corresponding platform moved forward and brought the food rewards within reach. Subjects were able to only reach food rewards on their side since the food item on the receiver’s side of the tray was more than two arm’s lengths (approximately 3 m) away from the pulling side. Monopolisation of both food items by the subject was impossible because pulling of the handle, repositioning to obtain the reward through the mesh and eating the reward took longer than it took the receiver to obtain its reward. Prior to the testing, we included a knowledge self-test to show that each subject could maximise its own benefit (Tan, Kwetuenda & Hare, 2015; Jensen, 2016b; Marshall-Pescini et al., 2016). In the self-test, subjects were tested individually and were presented with identical options as in the actual experiment (*i.e*., prosocial 1/1 and selfish 1/0, counterbalanced across platforms) but had the possibility to access both sides of the tray. In this case, choosing the 1/1 option would result in an extra reward, because they could reach and eat the food items at both sides. Only seven bonobos that chose the 1/1 option in 8 of 10 consecutive trials were considered to pass the self-test and were included as subjects in the actual experiment (see Table 1). All bonobos acted as receivers throughout the entire experiment. Therefore, although only seven bonobos acted as subjects, they participated with the different potential receivers, resulting in 49 unique (unidirectional) pairs (mean 7 ± 2 partners per subject).

The PCT was conducted during twelve different sessions, in which subjects could freely choose to participate. Therefore, the number of trials per session differed between sessions as individuals varied in their participation levels between sessions. Before each trial, the handles were pulled back and the platforms were baited. Trials started as soon as the experimenter, who was seated behind the apparatus, made the handles available by pushing the handles forward. In each trial, subjects could only choose one option because the space between both handles made it impossible for the bonobos to grab them with one hand to pull and because the other handle was pulled back as soon as the subject chose one option. To make sure that only one animal at a time could act as a subject, and to increase the chance of only testing one receiver, handles were only made available when one individual was present at the subject side and another individual sat in front of the receiver side of the apparatus. The experiment consisted of two types of sessions: test sessions and control sessions. Over ten test sessions, each bonobo had the possibility to act as a subject in maximal 20 trials with each receiver of the group (a maximum of 240 trials in total). In the two control sessions, the receiver side of the apparatus was positioned behind a plexiglass panel to block access to the food on the receiver’s side. Control trials were also freely accessible and each subject could participate in maximal 20 trials. The two control sessions were conducted after the ten test sessions to avoid that subjects would lose interest in the receiver’s side of the apparatus. The top/bottom position of the 1/1 option was randomised and counterbalanced across test and control trials. No trials were excluded from the analyses.

#### Experiment 2: Group service paradigm

**Figure 2:**
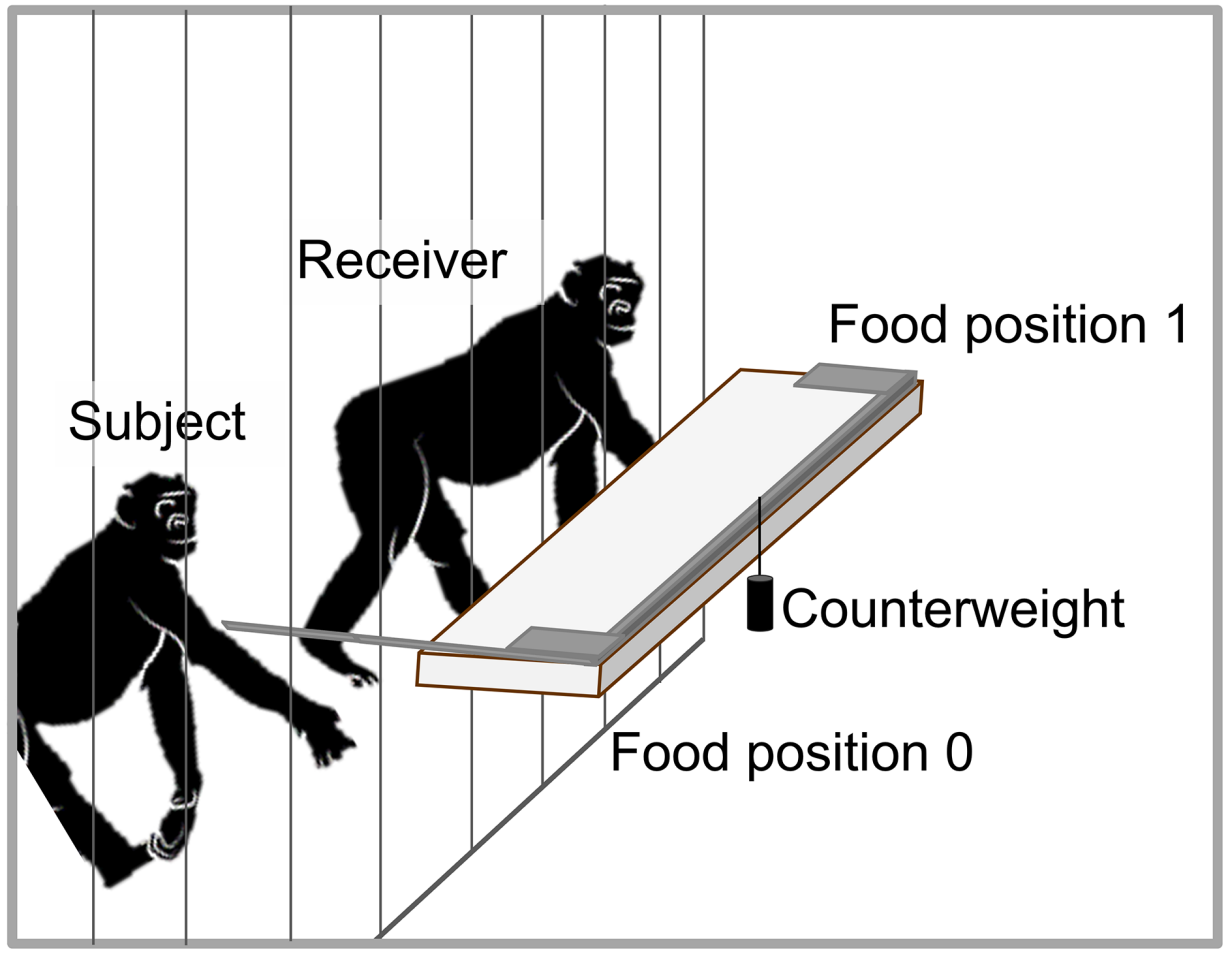
Schematic drawing of the GSP set-up. The handle can be pulled to bring the platform within reach. When the handle is released, the platform slides back due to the counterweight. Food can be placed on position 0 (motivational trials) and 1 (test trials). Food in position 1 can only be obtained by an individual if another individual pulls the handle and holds it in place.

We copied the setup of the original GSP studies (Burkart & van Schaik, 2013; Burkart et al., 2014). Subjects could choose to either pull and deliver a grape half to a group member or not pull and provide nothing (Fig. 2). The apparatus consisted of one slidable platform with a handle at one side of the platform. When a subject pulled the handle, the platform moved within reach. As soon as the subject released the handle, the platform slid back out of reach due to the attached counterweight. To deliver food to a receiver, a subject had to pull the handle and keep it in place. During test trials, the distance between the handle and the grape was more than two arm’s lengths, making it impossible for a subject to both pull and obtain the grape (cfr., Burkart & van Schaik, 2013; Burkart et al., 2014). The only way to obtain food was when another individual pulled and held onto the handle (0/1).

Prior to testing, we included a knowledge self-test. In this self-test, the distance between the handle and the grape was only one arm length, allowing subjects to pull and obtain the grape themselves. Subjects passed the self-test if they were able to pull the handle, hold it and obtain the grape in at least seven trials during one session (cfr., Burkart & van Schaik, 2013) (see Table 2). All bonobos acted as receivers throughout the entire experiment.

**Table 2:** Factors that influenced the likelihood of choosing the 1/1 option in the PCT.

Factor	Estimate	Standard error	*z*-value	*p*-value	Odds ratio	95 % CI	
						Lower	Upper
Condition	0.097	0.212	0.457	0.647	0.908	0.6	1.37
Position	−1.204	0.170	−7.094	<0.001	3.33	2.39	4.65

**Note:**

Variables are coded so that odds ratios exceed 1 for condition if actors were more likely to choose the 1/1 option when the receiver’s reward was blocked than when the receiver’s reward could be obtained by group members. Position was coded so that odds ratios larger than 1 indicate that actors were more likely to choose 1/1 option when positioned on the bottom platform of the set-up.

During the actual experiment, we alternated five test sessions with five control sessions (cfr., Burkart & van Schaik, 2013; Burkart et al., 2014). Each test session consisted of 70 test trials in which a grape half was placed on food position 1 (Fig. 2). Trials ended either when the grape was obtained, or after 1 min passed since the beginning of the trial. If the grape piece was not taken, the experimenter started the next trial by holding the grape up, attracting the bonobos’ attention and replacing the grape on food position 1. Control sessions were implemented to make sure that the bonobos did not pull simply to play or to explore the apparatus. Each control session consisted of 35 trials. Instead of placing a grape, the experimenter held up a stick, touched the apparatus at food position 1 and drew the bonobos’ attention verbally. To keep the individuals interested throughout the test and control sessions, every 6th trial a motivation trial was introduced. In motivation trials, a grape was placed on food position 0 and could be obtained by the pulling subject, resulting in a selfish outcome (cfr., Burkart & van Schaik, 2013; Burkart et al., 2014). Because of practical restrictions, we could not conduct the blocked control phase (phase V of the original GSP studies (Burkart & van Schaik, 2013; Burkart et al., 2014)).

### Coding and analyses

To determine whether bonobos behaved prosocially in the PCT, we ran a logistic regression analysis using a generalised linear mixed model (GLMM) with binomial error structure and logit link function using the R package “*lme4*” (Bates, Maechler & Bolker, 2014). The response variable was the binary variable that captured whether the prosocial 1/1 choice was chosen (coded as “1”) or not (coded as “0”). To investigate whether bonobos pulled more often in the test than control trials, we included ‘condition’ (control *vs* test trial) as fixed factor. We also added position as a fixed effect to determine whether the ‘position’ (top *vs* bottom) of the receiver’s reward influenced the choice of the subject. Subject identity was added as a random intercept effect to control for the non-independence of observations. We used likelihood ratio tests (R function *anova* with argument “test” set to “Chisq” (Dobson & Barnett, 2018)) to test the fixed effects by excluding each predictor at a time and comparing this reduced model to the respective full model using the function *drop1* (Barr et al., 2013). To assess the influence of possible collinearity between independent variables, the variance inflation factors (VIF) of each model were evaluated with the function *vif* of the R package “*car*” (Fox & Weisberg, 2019). None of the variables were rejected from the model (all VIF > 5) (O’Brien, 2007). We used diagnostic plots to examine assumptions of normality and homogeneity and tested uniformity and dispersion of the residuals using the “*DHARMa”* package (Hartig, 2020). To check whether our conclusions were robust to different forms of analyses, we also repeated the PCT analyses using Wilcoxon signed-ranks tests (see Supplemental Material).

To test whether bonobos behaved prosocially in the GSP, we assessed whether subjects pulled more often during the test *vs* the control trials using Wilcoxon signed-ranks test. Next, we analysed the trials in which subjects delivered food to group members, *i.e*., where the handle was pulled long enough to allow recipients to take the grape. All analyses were performed using R version 4.0.2 (R Core Team, 2016).

## Results

### Prosocial choice task

**Figure 3:**
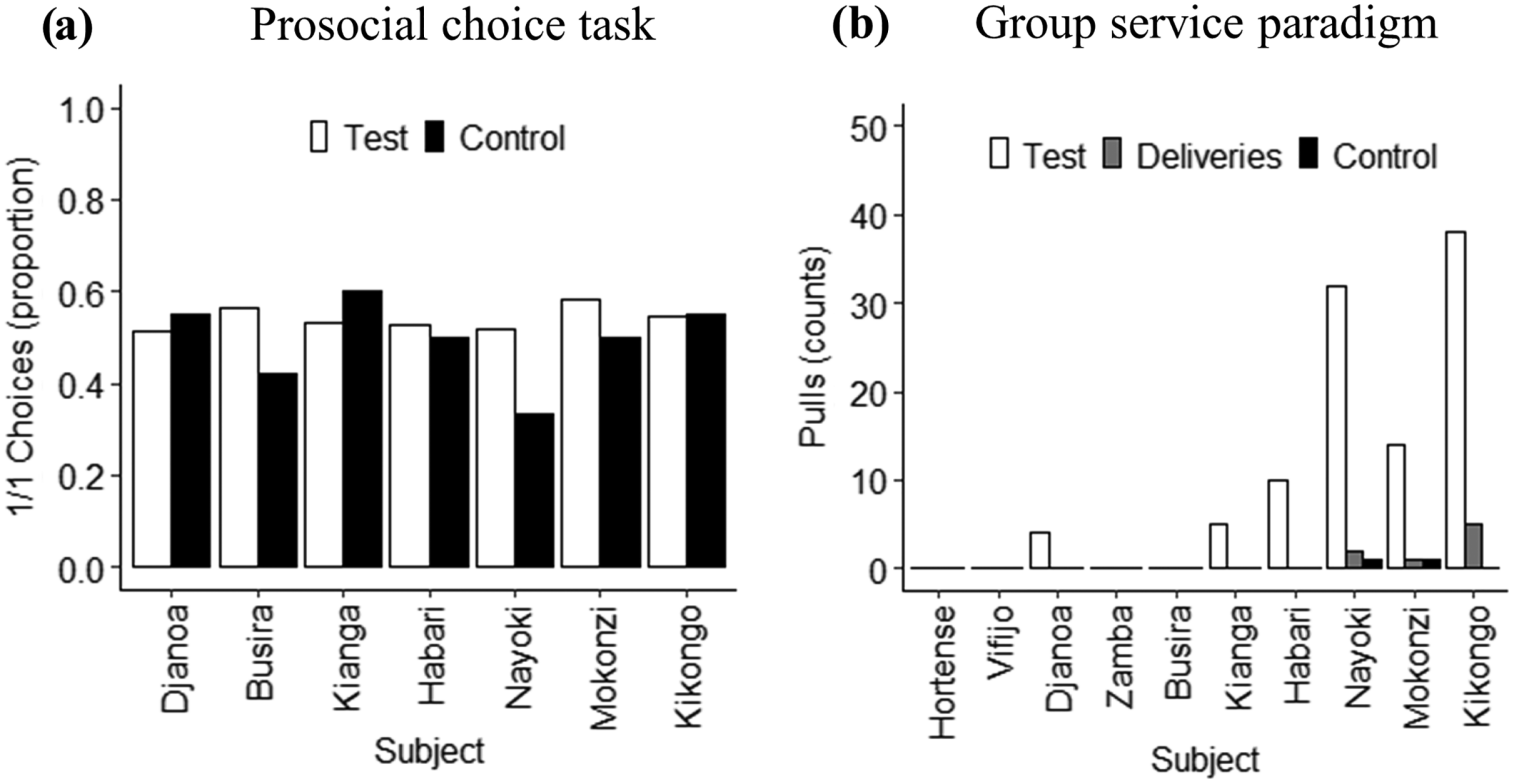
Prosociality results. (A) Proportion of 1/1 choices made by each subject in the PCT. Black bars represent control trials, white bars represent test trials. None of the subjects chose the 1/1 option more often in the test than the control trials (GLMM’s: all *p* > 0.05; see Table S1); (B) Total amount of pulls by each subject in the group service paradigm. Black bars represent control trials, grey bars represent deliveries and white bars represent test trials. Subjects pulled significantly more often during test than control trials (*p* < 0.05, Wilcoxon signed rank test).

**Figure 4:**
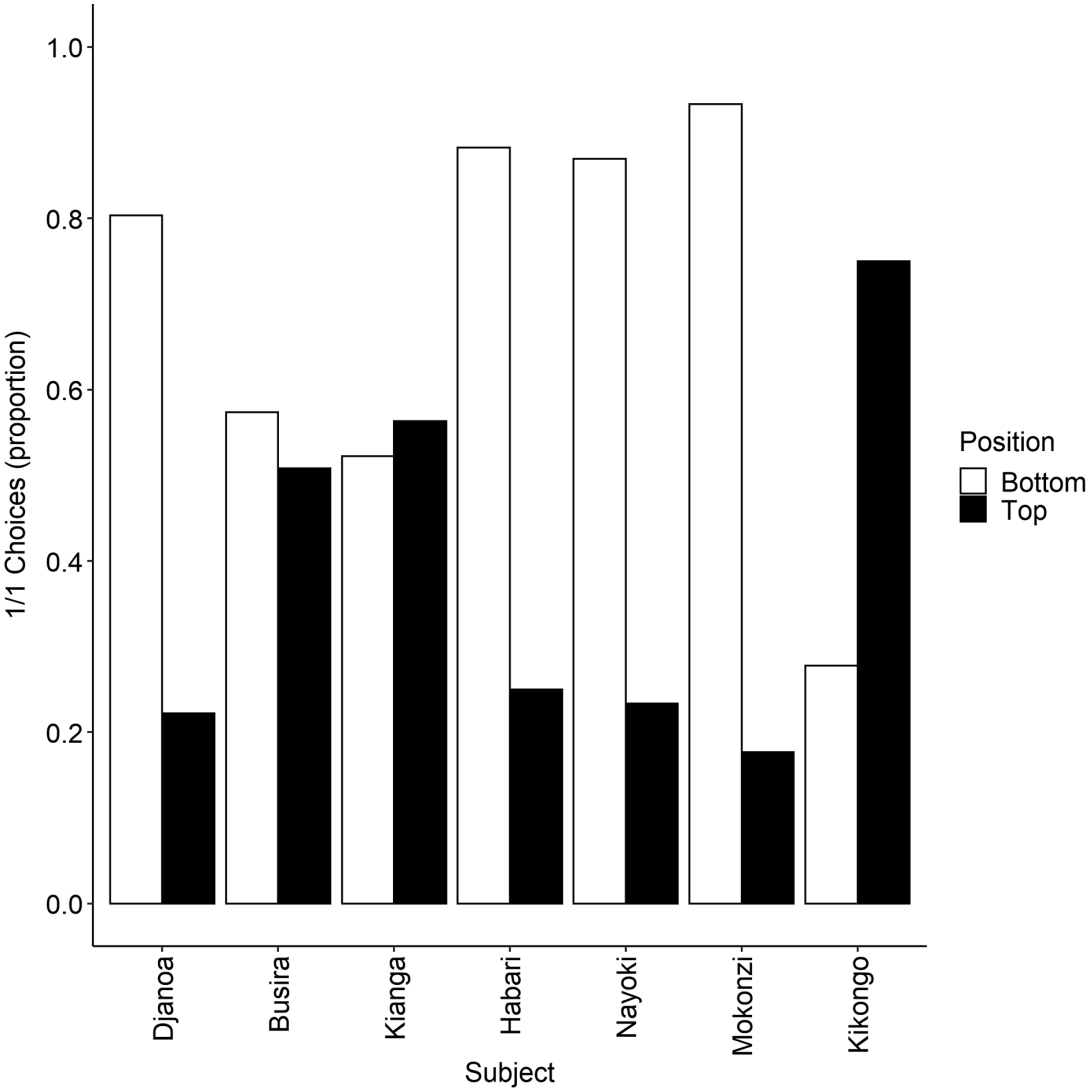
Proportion of 1/1 choices made by each subject in the PCT.

Bonobos chose the prosocial option on average in 53% (s.e. = 1.4) of the test trials (when receivers could obtain the reward) and in 49% (s.e. = 3.1) of the control trials (when no receiver could obtain the reward) (Fig. 3A). Overall, the logistic regression model showed that the likelihood of choosing the prosocial option was not significantly influenced by condition (χ^2^ = 0.21, df = 1, *p* = 0.65), but by position of the food reward (χ^2^ = 50.32, df = 1, *p* < 0.001) (Table 2). This means that subjects did not choose the prosocial option more often in the test than control trials and that the likelihood of choosing the prosocial option was affected by the position of the reward at the receiver’s side. Overall, subjects were more likely to choose the prosocial option when it was positioned on the bottom platform (Table 2, Fig. 4). Also at the individual level, none of the actors chose the 1/1 option more often in the test than control trials (all *p* > 0.05) (Fig. 4). We did find that for some individuals the likelihood of choosing the 1/1 option was significantly influenced by the position of the receiver’s reward, indicating that these individuals preferred one of both handles of the apparatus (for more information see Figure S1 in Supplemental Material).

### Group service paradigm

During motivation trials, all food items were taken. Two females monopolised the apparatus and obtained all the grapes. During the control and test sessions, bonobos rarely pulled the platform. Table 1 shows the number of pulls in test and control trials and the amount of food deliveries for each of the thirteen bonobos. Out of the ten bonobos, that had passed the training criterium, four bonobos never pulled the handle during the experiment. Out of the six subjects that did pull the handle, one adolescent and one juvenile also pulled once during a control trial. Most pulling was done by the adolescent female and two juvenile males (Fig. 3B). Overall, subjects pulled more often in the test than the control trials (test: mean = 10.3 ± 5.26; control: mean = 0.2 ± 0.16; *N* = 10, *Z* = 0.66, *p* = 0.04, Wilcoxon signed ranks test), suggesting that pulling was deliberate. Subjects pulled the handle in 29.4% of the test trials and in 1.1% of the control trials. In only 2% of all test trials (8 out of 350), however, grapes were successfully delivered to another individual. All successful deliveries were performed by the adolescent female and two juvenile males. One food item was delivered from the adolescent female to her mother, while seven deliveries happened between unrelated individuals (for more information see Supplemental Material).

## Discussion

Overall, the adult bonobos in this social group did not behave prosocially in two free choice group experiments. The adult bonobos were clearly motivated to obtain a reward for themselves in the PCT and GSP but they did not choose to provision partners above chance level; in the PCT, they did not pull more often in the test than the control sessions; and in the GSP, they never delivered food items. This suggests that in these food provisioning tasks, bonobos did not behave prosocially, but were rather indifferent to the gains of group members.

In the stacked platform PCT, bonobos did not choose the prosocial option more often in the test than the control trials. Our results therefore support previous studies that showed a lack of prosociality in bonobos in the horizontal and token PCT (Amici, Visalberghi & Call, 2014; Tan, Kwetuenda & Hare, 2015). During the test trials, the bonobos in our study chose the prosocial option in roughly half of the trials, which is higher than what a previous horizontal PCT study reported (±18%) but much lower than the levels in another horizontal PCT study (±79%) (Tan, Kwetuenda & Hare, 2015), which could be attributable to intergroup differences in prosocial tendencies between these bonobo populations. However, it is noteworthy that in the latter study, subjects chose the prosocial option in ±79% of the test and in ± 84% of the control trials, which is likely the result of local enhancement due to overtraining during the self-regard pre-test (Tan, Kwetuenda & Hare, 2015).

Our findings are also comparable with PCT studies that showed an absence of prosociality in chimpanzees (Silk et al., 2005; Jensen et al., 2006; Vonk et al., 2008; Claidière et al., 2015). Also in chimpanzees, the likelihood of choosing the prosocial 1/1 option was influenced by position of the reward, but not condition of the trials (Silk et al., 2005). One of the criticisms of the PCT is that it might be cognitively too demanding, leading to false results (Tan, Kwetuenda & Hare, 2015; Melis, 2018). This would suggest that subjects in our study understood how to obtain food for themselves, but not that they could deliver rewards to group members. However, the self-test phase showed that each of the subjects understood the consequences of each choice. The implementation of the stacked platform PCT in our study not only allowed for the knowledge self-test, it also controlled for any location bias, avoiding false positives due to local enhancement (Amici, Visalberghi & Call, 2014; Tan, Kwetuenda & Hare, 2015).

We found that in five out of seven bonobos, the prosocial option was more likely to be chosen when the receiver’s reward was positioned on a specific tray, meaning that these subjects developed a preference for one of the two handles. These choices seemed to be individualistic, *i.e*., not all seven subjects showed the same preference. Four of the bonobos chose the prosocial 1/1 option more often when positioned on the lower tray; one individual chose the prosocial 1/1 option more often when positioned on the upper tray, and two subjects did not show any preference for the upper or lower tray. The individual handle preference combined with the benefits of using the stacked platform PCT set-up (Tan, Kwetuenda & Hare, 2015), leads us to conclude that neither overtraining of the bonobos nor local enhancement explained the lack of prosociality in the PCT. Note that individual preferences for one handle over the other might have made it more difficult for the bonobos to show their potentially (non-)prosocial tendencies.

Using the GSP for the first time in bonobos, we found that there were no deliveries by adults, but subadult bonobos on several occasions delivered food to their group members. All subjects pulled more often in the test than control trials, suggesting that they pulled deliberately. Although subjects pulled the handle in 29.4% of the trials, food provisioning was low (2% over all five sessions and 2.02% during the last two sessions). This rate is much lower than reported for chimpanzees in the original GSP study (12% during the last two sessions (Burkart et al., 2014)). This suggests that bonobos in our study group behaved much less prosocially in the GSP compared to chimpanzees. Subjects needed to pull the handle long enough to deliver the grape to a conspecific. Similar to the capuchins and the macaques in the original GSP study, the majority of pulls by the bonobos did not result in a delivery (Burkart & van Schaik, 2013). In most events, subjects stopped pulling and released the handle before the receiver could obtain the reward. These results support the conclusion that while the bonobos were able to benefit group members, only subadults persisted in the pulling behaviour until the food was delivered in a limited amount of trials. One explanation for why the adult bonobos did not provide food to group members in this task, could be that behaving prosocially in the GSP creates inequity between subject and receiver. By refraining from providing food to group members, adult bonobos may have avoided the emergence of inequity, to which they might be averse (J. Verspeek & J.M.G. Stevens, 2021, in preparation). The motivation to provide benefits to others is then lower than the motivation to avoid inequity. Although a previous study found no conclusive evidence that bonobos showed aversion to inequity (Bräuer, Call & Tomasello, 2009), our own results in this study group show that the bonobos react to receiving less than a partner in a token exchange task and refuse to participate (J. Verspeek & J.M.G. Stevens, 2021, in preparation). Although more GSP and inequity studies are needed, this hypothesis is supported by the findings that primate species showing the lowest levels of proactive prosociality in the GSP (chimpanzees, macaques, capuchin monkeys, bonobos) also responded to inequity (Brosnan & de Waal, 2003; Van Wolkenten, Brosnan & De Waal, 2007; Brosnan et al., 2010; Takimoto, Kuroshima & Fujita, 2010; Massen et al., 2012; Hopper et al., 2014 but see Bräuer, Call & Tomasello, 2009), while species that showed relatively high levels of proactive prosociality in the GSP (*e.g*. callitrichids) were not found to be averse to inequity (reviewed in Brosnan & de Waal, 2014).

Overall, our study shows that bonobos did not take advantage of the opportunity to deliver food rewards to group members, even at little cost for themselves. Our findings are therefore comparable to previous food-based studies that tested for prosociality in the *Pan* species implementing food provisioning experiments in preselected pairs of chimpanzees (dyadic level: Silk et al., 2005; Jensen et al., 2006; Vonk et al., 2008; Amici, Visalberghi & Call, 2014; Tan, Kwetuenda & Hare, 2015; group level: Burkart et al., 2014; House et al., 2014 but see van Leeuwen et al., 2021). This seems surprising and controversial, since bonobos have been observed sharing food with related and unrelated individuals in the wild (*e.g*., White, 1994; Hohmann & Fruth, 1996; Hirata et al., 2010; Yamamoto, 2015) and behaved prosocially in several other experimental studies (*e.g*. Hare et al., 2007; Hare & Kwetuenda, 2010; Tan & Hare, 2013). We propose two possible explanations for these contrasting findings. First, in the majority of the food sharing events, food was acquired by stealing and tolerated theft (*i.e*. the owner of a food item does not facilitate taking but tolerates the recipient’s taking) (Jaeggi, Stevens & Van Schaik, 2010; Yamamoto, 2015), and shared food items were usually abundant or too large to consume by one individual. In situations with a surplus of food, owners can afford to pay a little cost by sharing only small portions of their food item (Yamamoto, 2015), while in food provisioning studies the limited food rewards may induce a more competitive context (Jaeggi, Burkart & Van Schaik, 2010). Second, the contrasting findings in the prosociality experiments in bonobos may be caused by an age effect. Previous experimental studies that reported prosociality in bonobos mainly included subadults and even juveniles in their helping and food sharing experiments (Hare et al., 2007; Hare & Kwetuenda, 2010; Tan & Hare, 2013). Additional instrumental helping studies that also reported prosocial tool and food transfers only tested juvenile, adolescent and young adult bonobos (Tan & Hare, 2017; Krupenye, Tan & Hare, 2018; Nolte & Call, 2021). Moreover, studies that replicated some of the experimental designs in a more diverse bonobo group (*i.e*. consisting of individuals of a wider age range and different backgrounds), failed to find the previously reported high levels of social tolerance, cofeeding and prosociality in bonobos (Jaeggi, Stevens & Van Schaik, 2010; Bullinger et al., 2013; Cronin, De Groot & Stevens, 2015) (for an overview of the ages of the study subjects see Table S4). Our data support this age effect hypothesis, since most pulling and prosocial acts in our study were done by subadults and juveniles. This corresponds with studies that suggested that younger bonobos may have higher other-regarding levels (Clay & de Waal, 2013a) and higher tolerance levels (Cronin, De Groot & Stevens, 2015), perhaps as a result of their lower development of inhibitory control (Clay & de Waal, 2013b). The lower inhibitory control could also explain why in the door-opening paradigm, subadults and juveniles behaved more prosocially (Hare & Kwetuenda, 2010) than older bonobos (Bullinger et al., 2013). Although the subadults were able to differentiate between the test and control trials in the GSP, their lower levels of inhibitory control might make them more prone to local enhancement when food is involved (test trials), thus resulting in the food deliveries. This study does not allow to differentiate prosociality from such alternative explanations for the pulling behaviour in subadults, because of the missing blocked-control (phase V in Burkart & van Schaik (2013); Burkart et al. (2014)). However, the very low levels of food deliveries do provide evidence for a low motivation to provision group members. Future studies with more diverse and a larger number of study subjects are needed to confirm our findings on the low levels of food provisioning in adult bonobos and to specifically address the possible age effect on bonobos’ prosocial behaviour. Also, although the GSP does not allow to investigate the motivation behind pulling behaviour, it is possible that the limited pulling by adult bonobos could have served as re-examinations of the task contingencies. After they started pulling, their self-inhibiting capacity might have allowed them to stop holding on the handle as soon as they realised that a conspecific would benefit from their action, hence avoiding food delivery and more frequent pulling. This would mean that the adult bonobos actively chose not to provide a resource to any of their group members.

An additional limitation of this study was that the experimental procedures involved high levels of manipulation by the experimenter. Although we aimed to use an open test setting that allowed free choice of participation, the current paradigms may have influenced naturally occurring patterns and therefore participation of certain individuals. Ideally, open group experiments should use an apparatus with automatic baiting to avoid any experimenter bias (*e.g*. (van Leeuwen et al., 2021)). Also, because the experiments were performed in a group setting, some pairs of subjects never participated. This could be the result of a lack of motivation to participate but is also likely that competition or interference by group members may have hindered participation of certain subjects. Only one receiver could obtain the reward, but we cannot rule out that the presence of multiple possible receivers in the testing room may have influenced the subject and receiver behaviour. Providing multiple apparatuses at the same time may overcome this reduced participation in a group setting (Cronin et al., 2017).

Despite the popular view of the prosocial bonobo, the implementation of both paradigms shows a lack of prosociality in food-based experiments in this group of bonobos. The adult bonobos did not show prosociality in a context where behaving prosocially did not incur a cost to the actor (PCT) and neither did they in a cognitively less demanding food provisioning task (GSP). By including the self-regard pre-tests and the counterbalanced trials in the stacked PCT set-up, we overcame the suggested constraints intrinsic to the PCT (Tan, Kwetuenda & Hare, 2015). In addition, although the GSP payoff is suggested to resemble instrumental helping paradigms (Tan, Kwetuenda & Hare, 2015), our findings contrast with the positive targeted helping of young bonobos (*e.g*., Hare & Kwetuenda, 2010; Tan & Hare, 2013; Tan, Ariely & Hare, 2017), while they do correspond with the negative helping results in older bonobo groups (Bullinger et al., 2013; Liebal et al., 2014).

Therefore, our results correspond with the predictions of the cooperative breeding hypothesis, as the non-cooperatively breeding bonobos show a very limited amount of proactive prosociality (Burkart et al., 2014). It also suggests that although bonobos do show signatures of self-domestication (Hare, Wobber & Wrangham, 2012; Hare, 2017), proactive prosociality might not be part of this domestication syndrome. Although the cooperative breeding hypothesis explains the negative proactive food provisioning results in independently breeding species like capuchin monkeys, macaques, bonobos, chimpanzees, it fails to explain the presence of reactive prosociality in these species (reviewed in Jensen (2016b); Marshall-Pescini et al. (2016)). In contrast to cooperative breeders, independent breeders do not need to proactively seek opportunities to provide food to others (Burkart & van Schaik, 2010). As a result they might need explicit signals of a need or a goal of others in order to show prosocial behaviour like helping or comforting (Jensen, 2016b). Therefore, rather than focusing on one prosocial behaviour and extrapolating the conclusions to explain prosocial behaviour as a whole, future studies should aim to investigate the multidimensional nature of prosocial behaviour in each species by including different pre-validated paradigms. Although additional research is needed to pinpoint whether the contrasting conclusions regarding bonobo prosociality are due to studying different facets of prosocial behaviour, or the age of the subjects, our study provides an important nuance in the existing field of prosociality research in bonobos.

## Conclusions

In contrast to the popular view of the prosocial and food sharing bonobo, we show that adult bonobos do not behave prosocially in two food provisioning paradigms. This suggests that proactive prosociality may not be part of the self-domestication syndrome in bonobos. Since food provisioning studies likely evoke competitive behaviour around the limited food rewards (Jaeggi, Burkart & Van Schaik, 2010), instrumental helping tasks or token variants of the prosociality paradigms could offer alternatives to study reactive prosocial behaviour in independently breeding species. Additional prosociality research in more diverse social groups is needed to expand our knowledge on the specific contexts that elicit prosociality in bonobos.

## Supplemental Information

10.7717/peerj.12849/supp-1 Supplemental Information 1 Group prosociality results.

10.7717/peerj.12849/supp-2 Supplemental Information 2 R code.

10.7717/peerj.12849/supp-3 Supplemental Information 3 Raw data.

10.7717/peerj.12849/supp-4 Supplemental Information 4 Example video PCT test and control trials.

10.7717/peerj.12849/supp-5 Supplemental Information 5 Example video GSP No pulling.

10.7717/peerj.12849/supp-6 Supplemental Information 6 Example video GSP Pulling that did not result in a delivery.

10.7717/peerj.12849/supp-7 Supplemental Information 7 Example video GSP test trials - delivery.

10.7717/peerj.12849/supp-8 Supplemental Information 8 Example video GSP control and motivational trials.

## Additional Information and Declarations

### Data Availability

The following information was supplied regarding data availability: The raw data is available in the Supplemental File.
