# Supplementary material for: Adult bonobos show no prosociality in both prosocial choice task and group service paradigm
Source: PeerJ. 2022 Feb 1;10:e12849. doi: 10.7717/peerj.12849 (PMC8815371; doi:10.7717/peerj.12849)
Supplement: Supplemental Information 3 [file peerj-10-12849-s003.docx]

Electronic Supplementary Material for

Adult bonobos show no prosociality in both prosocial choice task and group service paradigm

Experiment 1: Prosocial choice task

**Table S1** Factors that influenced the likelihood of choosing the 1/1 option in the PCT for each subject.

| Subject | Factor | Estimate | Standard error | z-value | p-value | Odds Ratio | 95% CI | |
| --- | --- | --- | --- | --- | --- | --- | --- | --- |
|  |  |  |  |  |  |  | Lower | Upper |
| Busira | Condition | 0.57 | 0.51 | -1.12 | 0.26 | 0.57 | 0.21 | 1.53 |
|  | Position | -0.26 | 0.37 | 0.7 | 0.48 | 1.29 | 0.63 | 2.65 |
| Djanoa | Condition | -0.28 | 0.61 | 0.45 | 0.65 | 1.32 | 0.40 | 4.35 |
|  | Position | -2.67 | 0.47 | 5.67 | <0.001 | 14.4 | 5.74 | 36.3 |
| Habari | Condition | 0.28 | 0.64 | -0.44 | 0.66 | 0.76 | 0.22 | 2.66 |
|  | Position | -3.12 | 0.52 | 6.02 | <0.001 | 22.8 | 8.23 | 63.0 |
| Kianga | Condition | -0.27 | 0.49 | 0.55 | 0.58 | 1.31 | 0.50 | 3.45 |
|  | Position | 0.17 | 0.34 | -0.49 | 0.62 | 0.85 | 0.43 | 1.65 |
| Kikongo | Condition | -0.37 | 0.72 | 0.51 | 0.61 | 1.45 | 0.35 | 5.99 |
|  | Position | 2.12 | 0.73 | -2.92 | 0.004 | 0.12 | 0.03 | 0.50 |
| Mokonzi | Condition | 1.48 | 1.27 | -1.17 | 0.24 | 0.23 | 0.02 | 2.75 |
|  | Position | -4.65 | 1.41 | 3.30 | 0.001 | 104 | 6.62 | 1646 |
| Nayoki | Condition | 0.82 | 1.63 | -0.51 | 0.61 | 0.44 | 0.02 | 10.2 |
|  | Position | -3.09 | 0.76 | 4.07 | <0.001 | 22.0 | 4.97 | 97.2 |
| Variables are coded so that odds ratios exceed 1 for condition if actors were more likely to choose the 1/1 option when the receiver’s reward could be obtained by group members than when the receiver’s reward was blocked. Position was coded so that odds ratios larger than 1 indicate that actors were more likely to choose 1/1 option when positioned on the bottom platform of the set-up. | | | | | | | | |

Additional analyses to confirm the results of the GLMM of the PCT study

To show that the results of the generalised mixed models are not the result of underpowered analyses, we conducted additional analyses. We used Wilcoxon signed rank tests to compare the preferences between and within conditions. We found that subjects did not prefer the prosocial 1/1 to the selfish 1/0 option in the test nor the control trials (test: mean = 0.53 ± 0.02; self: mean = 0.47 ± 0.02; N = 7, Z = -1.54, *p* = 0.108; control: mean = 0.49 ± 0.03; self: mean = 0.51 ± 0.03; N = 7, Z = 0, *p* = 1, Wilcoxon signed-ranks). Also, the proportion of prosocial choices 1/1 did not differ between the test and control trials (N = 7, Z = -0.76, *p* = 0.447, Wilcoxon signed-ranks test). The subjects’ preferences did not differ between the first 20 and the last 20 test trials (first: mean = 0.44 ± 0.04; last: mean = 0.59 ± 0.05; N = 7, Z = 1.90, *p* = 0.06, Wilcoxon signed rank test). These results show that subjects did not prefer the prosocial option to the selfish option in both the test and control trials. Although the GLMM results show that on a trial basis, some of the subjects preferred one of both handles, the position of the prosocial option was counterbalanced between the top and bottom platform, overcoming any false positives due to the handle preference. Therefore, these results show that bonobos did not behave prosocially in the PCT.

We also ran an additional GLMM, which was identical to the other model but also included the interaction term between condition and position. The logistic regression model showed that the likelihood of choosing the prosocial 1/1 option was significantly influenced by the interaction term between condition and position (χ² = 7.78, df = 1, *p* = 0.005). The model showed that subjects’ were more likely to choose the bottom handle than the top handle in the test and control trials (control: p < 0.001; test: p < 0.001) and that the odds of doing so were bigger in the control than the test trials (Table S2).

| Factor |  | Probability | Standard error | z-value | p-value | Odds ratio | 95 % CI | |
| --- | --- | --- | --- | --- | --- | --- | --- | --- |
|  |  |  |  |  |  |  | Lower | Upper |
| Condition |  |  |  |  |  |  |  |  |
| Test | Bottom | 0.660 | 0.031 | 5.191 | < 0.001 | 2.63 | 1.83 | 3.8 |
|  | Top | 0.42 | 0.03 |  |  |  |  |  |
| Control | Bottom | 0.780 | 0.054 | 5.336 | < 0.001 | 9.57 | 4.18 | 22.0 |
|  | Top | 0.270 | 0.031 |  |  |  |  |  |
| Position was coded so that odds ratios larger than 1 indicate that actors were more likely to choose 1/1 option when positioned on the bottom platform of the set-up. | | | | | | | | |

**Table S2** Factors that influenced the likelihood of choosing the 1/1 option in the PCT

Pulling behaviour in the first 20 test trials

In the PCT study, we included much more test than control trials. To determine whether this may have influenced our results, we conducted the same analyses only including the first 20 trials of each subject. For Mokonzi only 12 trials were included as he only participated in 12 test trials. Bonobos chose the prosocial 1/1 option on average 52% (s.e. = 1.8) in the first 20 test trials. The logistic regression model showed that the likelihood of choosing the prosocial 1/1 option was significantly influenced by the interaction between condition and position (χ² = 10.33, df = 1, *p* = 0.001). Post-hoc analysis showed that in the control trials, subjects were more likely to choose the lower handle than the upper handle (p < 0.001), while in the test trials the bonobos did not choose one handle more often than the other (p = 0.18).


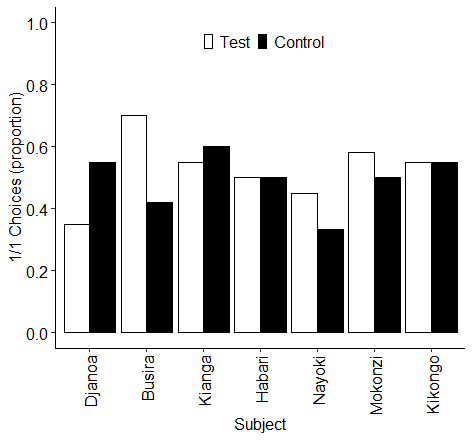


**Figure S1.** Proportion of 1/1 choices made by each subject in all control trials and the first 20 test trials of the PCT. Black bars represent control trials, white bars represent test trials.

Experiment 2: Group service paradigm

*Pulling behaviour across the five sessions*


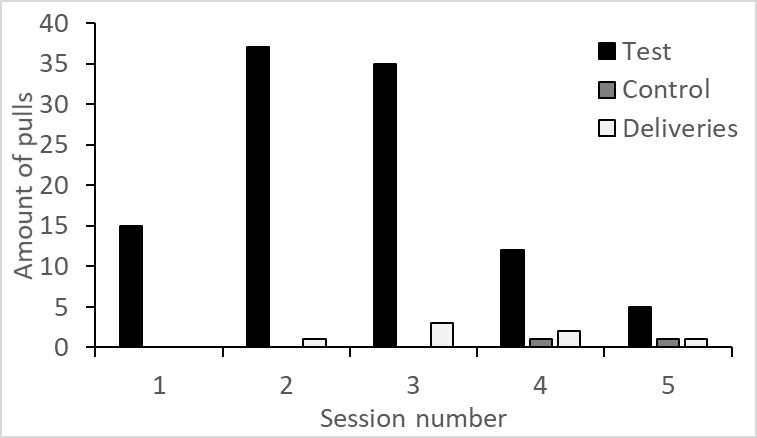


**Figure S2.** Number of pulls in test and control session and the number of deliveries across the five sessions. Black bars represent pulls in the test sessions, grey bars represent pulls in the control sessions and white bars represent food deliveries.

*Distribution of successful food deliveries*

| **Table S3.** Distribution of successful food deliveries for each dyad (food given vs received) | | | | | | | | | | | | | | | |
| --- | --- | --- | --- | --- | --- | --- | --- | --- | --- | --- | --- | --- | --- | --- | --- |
|  | DJ | KG | BY | HO | HB | BS | ZA | NY | VI | *MZ* | *KK* | *MO* | *SA* | Total given |  |
| DJ | - | 0 | 0 | 0 | 0 | 0 | 0 | 0 | 0 | 0 | 0 | 0 | 0 | 0 |  |
| KG | 0 | - | 0 | 0 | 0 | 0 | 0 | 0 | 0 | 0 | 0 | 0 | 0 | 0 |  |
| BY | 0 | 0 | - | 0 | 0 | 0 | 0 | 0 | 0 | 0 | 0 | 0 | 0 | 0 |  |
| HO | 0 | 0 | 0 | - | 0 | 0 | 0 | 0 | 0 | 0 | 0 | 0 | 0 | 0 |  |
| HB | 0 | 0 | 0 | 0 | - | 0 | 0 | 0 | 0 | 0 | 0 | 0 | 0 | 0 |  |
| BS | 0 | 0 | 0 | 0 | 0 | - | 0 | 0 | 0 | 0 | 0 | 0 | 0 | 0 |  |
| ZA | 0 | 0 | 0 | 0 | 0 | 0 | - | 0 | 0 | 0 | 0 | 0 | 0 | 0 |  |
| NY | **1** | 0 | 0 | 0 | 0 | 0 | 0 | - | 0 | 0 | 0 | **1** | 0 | 2 |  |
| VI | 0 | 0 | 0 | 0 | 0 | 0 | 0 | 0 | - | 0 | 0 | 0 | 0 | 0 |  |
| *MZ* | **1** | 0 | 0 | 0 | 0 | 0 | 0 | 0 | 0 | - | 0 | 0 | 0 | 1 |  |
| *KK* | 0 | 0 | 0 | 0 | **2** | 0 | 0 | **3** | 0 | 0 | - | 0 | 0 | 4 |  |
| *MO* | 0 | 0 | 0 | 0 | 0 | 0 | 0 | 0 | 0 | 0 | 0 | - | 0 | 0 |  |
| *SA* | 0 | 0 | 0 | 0 | 0 | 0 | 0 | 0 | 0 | 0 | 0 | 0 | - | 0 |  |
| **Total received** | 2 | 0 | 0 | 0 | 1 | 0 | 0 | 3 | 0 | 0 | 0 | 1 | 0 | 7 |  |
| Subjects are ordered according to their dominance rank. HO= Hortense, VI = Vifijo, ZA = Zamba, KK = Kikongo, BY = Banya, MO = Mokonzi, DJ = Djanoa, HB = Habari, NY = Nayoki, BU = Busira, SA = Sanza, KI = Kikongo, MK = Moko | | | | | | | | | | | | | | | |

*Presence of individuals in the testing room throughout the experiment*

To investigate whether the limited amount of pulls and successful deliveries was not result of the absence of possible receivers, we collected data on the amount of individuals in the testing room throughout the experiment. We used instantaneous group scan sampling with a fixed time interval of five minutes and collected data on the location of each of the individuals. For each instantaneous scan sample, we calculated the proportion of individuals that was present in the testing room. We found that in each scan sample during the test trials, at least 30% of the individuals, i.e. 4 potential participants, were present in the testing room (Figure S3). These data show that although potential receivers were present, subjects were not motivated to donate food items.


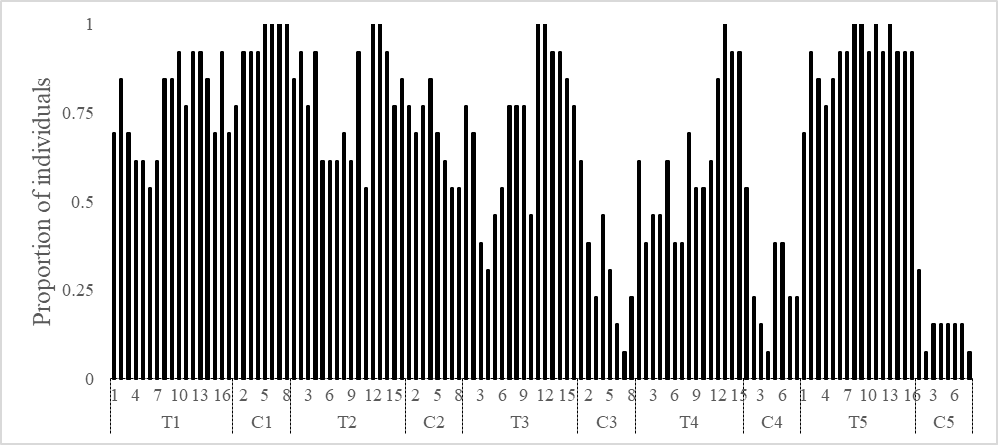


**Figure S3** Proportion of individuals that was present during each instantaneous scan sample (T = test session, C = control session).

Overview of the published experimental prosociality studies in bonobos

| **Table S4.** Summary of prosociality and food sharing experiments. Task refers to the kind of paradigm that was used. Result summarises the findings of the study. Prosocial reports whether the study found a positive or negative result. | | | | | | | |  |
| --- | --- | --- | --- | --- | --- | --- | --- | --- |
| **Study** | **Task** | **Sample size** | **Age range** | **Mean age** | | **Result** | **Prosociality** | |
| Hare et al. (2007) | Dyadic co-feeding^a^ | 20 | 5 - 22 | 9.6 ± 5.7 | | Tolerant cofeeding | + | |
| Hare & Kwetuenda (2010) | Door-opening paradigm^a^ | 8 | ° | | ° | Food sharing with group members | + | |
| Tan & Hare (2013) | Door-opening paradigm^a^ | 14 | 4 - 12 | 7.5 ± 2.5 | | Food sharing with strangers | + | |
| Tan et al. (2017) | Helping task^a^ | 16 | 5 - 15 | 9.2 ± 3.1 | | Prosocial helping | + | |
| Krupenye et al. (2018) | Helping task^a^ | 12 | 5 - 15 | 10.2 ± 3.2 | | Food transfer | + | |
| Nolte & Call (2021) | Helping task | 6 | 5- 16 | 12.2 ± 4.5 | | Object transfer | + | |
| Jaeggi et al. (2010) | Group co-feeding | 9 | 10 - 30 | 21.0 ± 7.9 | | Limited cofeeding | - | |
| Bullinger et al. (2013) | Door-opening paradigm | 6 | 6 - 28 | 15.0 ± 7.7 | | Prefer to feed alone | - | |
| Amici, Visalberghi, et al. (2014) | Lateral PCT | 9 | ^+^ | | ^+^ | No prosociality | - | |
|  | Token PCT | 9 | ^+^ | | ^+^ | No prosociality | - | |
| Tan et al. (2015) | Lateral PCT^a^ | 10 | 6 - 11 | 8.2 ± 1.8 | | No prosociality | - | |
|  | Lateral PCT^a^ | 9 | 6 - 11 | 8.4 ± 2.1 | | No prosociality | - | |
| Cronin et al. (2015) | Group co-feeding | 8 | 5 - 27 | 13.1 ± 6.9 | | Limited cofeeding | - | |
| Krupenye et al. (2018) | Helping task^a^ | 18 | 3 - 15 | 8.4 ± 3.3 | | No object transfer | - | |
| This study: experiment 1 | Stacked PCT | 13 | 2 - 41 | 15.8 ± 11.2 | | No prosociality | - | |
| This study: experiment 2 | GSP | 13 | 2 - 41 |  |  | No prosociality | - | |
| °Not provided but similar subjects as Hare et al. (2007) and Tan & Hare (2013) | | | | | | | |  |
| ^+^Not provided but some similar subjects as Bullinger et al. (2013) | | | | | | | |  |
| ^a^The majority of the tested dyads involves at least one adolescent or even juvenile | | | | | | | |  |
